# New Strategies and Combinations to Improve Outcomes in Immunotherapy in Metastatic Non-Small-Cell Lung Cancer

**DOI:** 10.3390/curroncol29010004

**Published:** 2021-12-23

**Authors:** Lucy Corke, Adrian Sacher

**Affiliations:** 1 Princess Margaret Cancer Centre, University Health Network, Toronto, ON M5G 2C1, Canada; lucy.corke@uhn.ca; 2 Department of Immunology, University of Toronto, Toronto, ON M5S 1A1, Canada

**Keywords:** non-small-cell lung cancer, immunotherapy, tumor microenvironment

## Abstract

Immune checkpoint inhibitors have transformed the treatment of metastatic non-small-cell lung cancer, yielding marked improvements in survival and the potential for durable clinical responses. Primary and acquired resistance to current immune checkpoint inhibitors constitute a key challenge despite the remarkable responses observed in a subset of patients. Multiple novel combination immunotherapy and adoptive cell therapy strategies are presently being developed to address treatment resistance. The success of these strategies hinges upon rational clinical trial design as well as careful consideration of the immunologic mechanisms within the variable tumor immune microenvironment (TIME) which underpin resistance to immunotherapy. Further research is needed to facilitate a deeper understanding of these complex mechanisms within the TIME, which may ultimately provide the key to restoring and enhancing an effective anti-tumor immune response. This review aims to provide an introduction to some of the recent and notable combination immunotherapy and cell therapy strategies used in advanced non-small-cell lung cancer (NSCLC), and the rationale for their use based on current understanding of the anti-tumor immune response and mechanisms of resistance within the TIME.

## 1. Introduction

Lung cancer is the leading cause of cancer-related death worldwide. Immune checkpoint inhibitors have revolutionized the treatment of metastatic non-small-cell lung cancer (mNSCLC), with marked improvement in overall survival and the potential for durable clinical benefit. However, long-term disease control is only seen in approximately 15–20% of patients and extensive investigation has yet to yield a definitive biomarker of durable response to existing immunotherapies [1]. There is growing understanding that the environment in which tumor and immune cells interact (the tumor immune microenvironment (TIME)) is crucial in determining therapeutic outcomes. Furthermore, improved understanding of the myriad complex interactions of immune, tumor and stromal cells within the TIME and draining lymph nodes has the potential to guide the rational development of novel immunotherapeutic combinations as well as identify mechanisms of primary and acquired resistance to existing immunotherapies. This review will utilize our current understanding of the TIME and the mechanisms involved in the induction of an effective anti-tumor immune response as a framework to understand the rationale as well as potential of novel combination immunotherapeutic and cell therapy strategies in mNSCLC.

## 2. The Tumor Immune Microenvironment (TIME)

**Figure 1:**
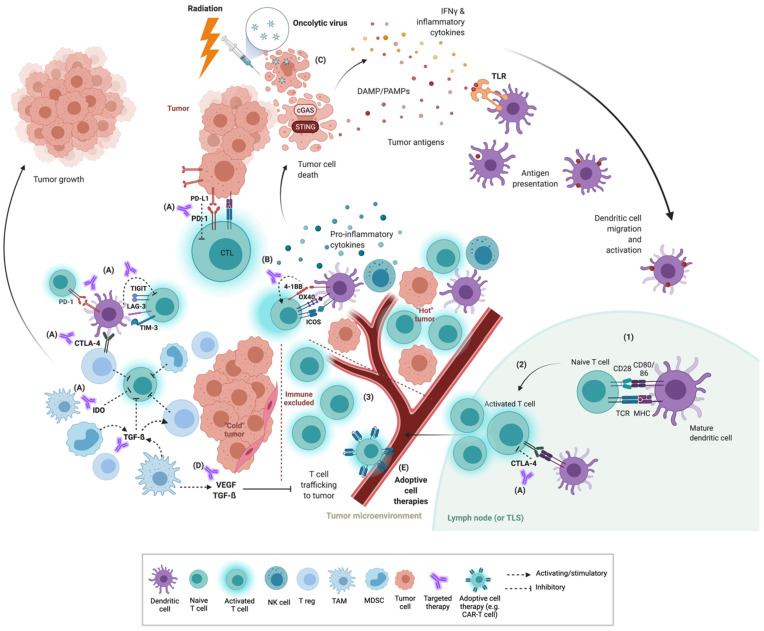
T-cell tumor response and interactions within the tumor immune microenvironment (TIME). Priming of T cells begins with presentation of tumor antigen to naïve T cells via antigen-presenting cells within draining lymph nodes (and potentially in tumor lymphoid structures, TLS) (1). Activation occurs when T-cell receptors (TCR) recognize their specific peptide antigen presented by MHC and in the presence of co-stimulation signaling between CD28 and CD80/86, with additional co-stimulation from inflammatory cytokines (2). Activated T cells undergo expansion and then traffic to the tumor where they can engage in direct cytotoxicity against tumor cells presenting cognate antigen via MHC. Tumor immune microenvironments can be conceptualised as “hot”, “cold” or “immune excluded” (3). “Hot” or inflamed TIME contain abundant CTLs and other immune cell populations (dendritic cells, NK cells) associated with anti-tumor immunity and expression of inhibitory checkpoints, and thus are more likely to respond to checkpoint inhibition resulting in tumor cell death. “Cold” tumors by contrast contain more immunosuppressive cell populations such as Tregs, TAMs, MDSCs. Immune-excluded tumors contain inflammatory immune infiltrate which is unable to penetrate into the TIME due to stromal populations and secreted factors such as TGF-β and VEGF. These latter two tumor environments are poorly responsive to checkpoint inhibition alone and new combination therapies are being investigated. Immunotherapeutic approaches include (**A**) blockade of inhibitory checkpoints, (**B**) stimulation of co-stimulatory checkpoints, (**C**) priming strategies to increase effective T-cell activation through stimulation of the innate immune system (e.g., radiation, oncolytic viral therapy and STING pathway agonists), (**D**) targeting immunomodulators (e.g., VEGF, TGF-β) and (**E**) introduction of primed or engineered T cells via adoptive cell therapy. CTL, cytotoxic T lymphocyte; DAMP, damage-associated molecular patterns; IDO, indole 2,3-dioxygenase; LAG-3, lymphocyte-activation gene 3; MDSC, myeloid-derived suppressor cell; MHC, major histocompatibility complex; PAMP, pathogen-associated molecular patterns; STING, stimulator of interferon genes; TAM, tumor-associated macrophage; TGF-β, transforming growth factor beta; TIGIT, T-cell immunoreceptor with Ig and ITIM domains; TIM-3, T-cell immunoglobulin and mucin-domain-containing molecule 3; TLR, Toll-like receptor; VEGF, vascular endothelial growth factor. Adapted from “Tumor-Specific T cell Induction and Function”, by BioRender.com (2020). Retrieved from https://app.biorender.com/biorender-templates, accessed on 10 September 2021.

The TIME represents a highly variable environment composed of multiple immune cell populations as well as stromal and tumor cells. Its composition reflects the complex interplay between the tumor and the immune system, offering a unique insight into the degree to which a functional immune response exists in a given tumor. T cells and natural killer (NK) cells are classically regarded as the primary workhorses of anti-tumor immunity, as their priming and activation are critical for anti-tumor response. Complex interactions exist between these and other immune cell populations, tumor and stromal cells, which all work to resist or promote the overall response (Figure 1).

An effective anti-tumor immune response hinges upon appropriate priming of T cells capable of recognizing and killing tumor cells. Priming begins with the presentation of tumor antigens to naïve T cells via antigen-presenting cells (APCs) within the local draining lymph nodes. T-cell receptors (TCR) on CD4+ and CD8+ T cells recognize their specific peptide antigen when presented by MHC but require additional costimulatory signals in order to activate. The interaction between CD28 on T cells and its ligands CD80/CD86 on the APC constitutes the key co-stimulatory signal required for T-cell priming and represents the archetypal example of co-stimulation. Additional stimulation by inflammatory cytokines is also required for CD8+ T-cell activation and adoption of a T-effector phenotype. Activated T cells subsequently undergo clonal expansion, translocation to the TIME and may potentially engage in direct cytotoxicity against cancer cells displaying their cognate antigen. However, failure at the priming stage may lead to T-cell anergy or differentiation in a regulatory T-cell phenotype (Treg).

The functional tumor-killing potential of specific immune cell populations (e.g., T cells and NK cells) must be considered within the complex and dynamic cellular milieu of the TIME in which they must operate. The TIME structure is underpinned by a network of tumor cells interacting with multiple stromal cell populations as well as the extracellular matrix [2]. These elements may serve to modulate immune cell activity either through direct interaction, secretion of soluble factors or by preferentially attracting specific immune cell populations via chemotaxis. The immune cell populations present in the TIME are potentially varied and may contain both immune cell populations with tumor-killing potential as well as multiple immune cell populations that may alternatively promote or suppress immune cell activity [3]. The varied composition of the TIME has broadly been conceptualized into three categories — inflamed (“hot”), non-inflamed (“cold”) and immune excluded (Figure 1).

An inflamed TIME is characterized by an active immune infiltrate with an abundance of cytotoxic T lymphocytes as well as other immune cell populations associated with anti-tumor immunity including dendritic cells (DC), NK cells and B cells. Tertiary lymphoid structures as well as plasma cells may also be present and support an anti-tumor immune response [4]. In contrast, a non-inflamed tumor is associated with an immunosuppressive TIME with minimal immune infiltrate or potentially tolerogenic immune cell populations such as Tregs, tumor-associated macrophages (TAMs) and myeloid-derived suppressor cells (MDSCs). Immune-excluded tumors often contain an active immune infiltrate which is displaced and unable to penetrate into the TIME likely secondary to immuno-modulatory stromal populations and secreted factors (e.g., TGF-β). Importantly, each of these immune categories should be considered a distinct entity produced by complex and varied immunologic mechanisms [5].

Careful study of the TIME has significant potential to reveal key mechanisms which underpin the interactions between tumor and immune cells resulting in the aforementioned immunologic states particularly in mNSCLC. It is precisely the nature of these pre-existing interactions that determines both the composition of the TIME as well as the potential success or failure of a given immunotherapeutic intervention. The presence of an active immune infiltrate with high-expression of co-inhibitory molecules such as PD-1/PD-L1 associated with an inflamed TIME has been heavily associated with response to PD-1 inhibitors in mNSCLC [6,7]. In contrast, PD-1 inhibitors are thought to be less effective in mNSCLC patients with a non-inflamed TIME likely secondary to the absence of an immune infiltrate capable of recognizing and eliminating tumor cells in response to downregulation of co-inhibitory signaling through PD-1 [8]. The development of effective and rational combination immunotherapy capable of yielding improved clinical efficacy beyond existing PD-1/PD-L1 inhibitor therapy hinges upon understanding these immunologic states, the mechanisms that underpin them, and the appropriate targets that can unleash a potent anti-tumor immune response in these specific immunologic contexts.

## 3. Co-Inhibitory and Co-Stimulatory Checkpoints

### 3.1. Co-Inhibitory Molecules

Activated T cells express a number of co-inhibitory molecules on their surface that contribute to sustaining or suppressing a cell-mediated immune response. CTLA-4 and PD-1 are both inhibitory checkpoint molecules expressed on multiple immune cells including activated T cells. CTLA-4 binds CD80/86 with much higher affinity than CD28 and inhibits activation of T cells. Binding of PD-1 to its ligand PD-L1, which can be expressed on both tumor and immune cells, also results in inhibition of T-cell activation. Blockade of PD-1/PD-L1 and CTLA-4 thus unleashes activated effector T cells to drive anti-tumor response within the TIME. There exist multiple additional co-inhibitory molecules (e.g., TIGIT, TIM-3, and LAG-3) which are potentially susceptible to therapeutic targeting. The development of novel combinations of drugs targeting co-inhibitory molecules is presently proceeding in earnest with the goal of improving upon the established efficacy of PD-1/PD-L1 inhibitor therapy in mNSCLC.

The use of PD-1/PD-L1 inhibitors transformed the mNSCLC treatment landscape and has demonstrated the ability to induce durable clinical benefit albeit in a subset of patients. The use of pembrolizumab (PD-1 inhibitor) in mNSCLC led to 5 year survival rates of 23% for treatment-naïve patients and 15% for those previously treated [1]. In patients with PD-L1 expression of ≥50%, 5 year survival rates improved to 32%, compared to 16% with standard platinum-doublet chemotherapy [9]. Atezolizumab is a PD-L1 inhibitor that also demonstrated improved survival compared to chemotherapy in patients with high PD-L1 expression, but not in those with lower expressions of PD-L1 [10]. Cemiplimab is another PD-1 inhibitor recently approved after demonstrating survival benefit, again in those patients with PD-L1 expression greater than 50% [11]. PD-1 inhibitor nivolumab demonstrated benefit against chemotherapy in previously treated squamous and non-squamous patients [12], but interestingly failed to show significant benefit in first-line patients with PD-L1 expression >5% [13]. Taken together, these findings provide a compelling rationale for the efficacy of PD-1 inhibitors in mNSCLC particularly with an inflamed TIME as well as the use of PD-1 inhibitors as a backbone for the development of novel immunotherapy combinations.

Ipilimumab is a CTLA-4 inhibitor which has been evaluated in combination with PD-1 inhibitors in mNSCLC. Ipilimumab-nivolumab, either alone or in combination with chemotherapy, has demonstrated survival benefit compared to chemotherapy alone [14,15]. The benefit of this combination immunotherapy appears consistent across PD-L1 status and may also be particularly beneficial in patients with treated brain metastases [16]. The recent KEYNOTE-598 trial evaluated ipilimumab-pembrolizumab in mNSCLC patients with PD-L1 ≥ 50% and failed to demonstrate improved survival compared to pembrolizumab alone [17]. It thus remains unclear as to which mNSCLC patients may derive improved clinical benefit from combination therapy with ipilimumab-nivolumab as opposed to PD-1 inhibitor therapy either alone or in combination with chemotherapy. There exists significant opportunity to define additional biomarkers in this context beyond PD-L1 expression alone to better identify patients that benefit from this combination. In particular, combination therapy with CTLA-4 may uniquely synergize with radiotherapy [18]. Ongoing trials using combinations of CTLA-4, PD-1 and other agents can be found in Table S1.

T-cell immunoreceptor with Ig and ITIM domains (TIGIT) is a member of the CD28 family and potently inhibits innate and adaptive immunity through a number of mechanisms. TIGIT inhibits NK cell-mediated tumor killing and suppresses CD8+ T-cell activation as well as cytotoxicity [19,20]. TIGIT is co-expressed with PD-1 on exhausted T cells providing a compelling rationale that dual blockade of TIGIT and PD-1 can restore T-cell immunity [21]. A phase 1 study of vibostolimab (anti-TIGIT) in mNSCLC patients previously treated with ICI demonstrated tolerable toxicity and modest anti-tumor activity. ORR was 7% with vibostolimab alone and 5% when combined with pembrolizumab; however, the combination improved median DOR from 9 to 13 months [22]. Tiragolumab (anti-TIGIT) was recently evaluated in a randomized phase 2 trial in combination with atezolizumab in untreated mNSCLC with PD-L1 ≥ 1%. Tiragolumab-atezolizumab provided a modest but statistically significant improvement in median PFS compared to atezolizumab alone, 5.6 months versus 3.9 months [23]. Interestingly, PFS benefit was most pronounced in those patients with high PD-L1 expression [24]. Phase 3 trials are underway in both front-line and later-line settings and these will hopefully highlight the patient subsets most likely to respond to this promising combination (Table S1).

Lymphocyte-activation gene 3 (LAG-3) is another co-inhibitory molecule expressed on T cells that binds MHC-II and inhibits activation of CD4+ cells. Preclinical studies have demonstrated co-expression of LAG-3 with PD-1 on exhausted T cells and that combined blockade results in synergistic anti-tumor immune activity [25,26]. Early-phase studies of eftilagimod alpha (IMP321), a soluble LAG-3 protein, demonstrated response in melanoma when combined with pembrolizumab [27] and there are encouraging initial results from a phase 2 study of the combination in mNSCLC with ORR reaching 47% and responses seen across PD-L1 expression levels [28]. Relatlimab is a LAG-3 antibody investigated in multiple solid tumors in combination with a range of other agents. Several other LAG-3 antibodies, fusion proteins and bi-specific molecules are also being evaluated in combination with other checkpoint inhibitors in mNSCLC (Table S1).

T-cell immunoglobulin and mucin-domain-containing molecule 3 (TIM-3) is expressed on tumor and immune cells and interaction with its ligands induces T-cell inhibition. Upregulation of TIM-3 on TILs is prognostic of poor outcome in multiple different cancers including NSCLC [29]. Preclinical work has suggested that blocking TIM-3 expression results in proliferation of T cells and cytokine production and demonstrates synergistic effect when combined with PD-1 blockade [30]. The phase 1 AMBER trial of TSR-022 (anti-TIM3) in combination with a PD-1 inhibitor reported partial responses in 4 of 31 mNSCLC patients who had previously progressed on PD-1/PD-L1 inhibitors [31]. Additional early-phase trials in solid tumors have suggested similar early signals of activity [32] (Table S1).

Indole 2,3-dioxygenase (IDO) is the rate-limiting enzyme involved in the conversion of tryptophan to kynurenine which promotes Treg and MDSC activity as well as suppresses T-effector cell activity. Multiple phase II studies of IDO1 inhibitors in combination with PD-1 inhibitors have suggested promising activity of this combination in multiple solid tumors [26]. A pivotal phase III trial of epacadostat (IDO1 inhibitor) and pembrolizumab in treatment-naïve metastatic melanoma has recently failed to demonstrate PFS benefit [33]. This negative result has lessened enthusiasm for this drug class and many associated trials have been halted. The trial failure has largely been attributed to the unselected nature of the study population thus making the case for more in-depth biomarker analysis to select patients for such trials. Ongoing trials with epacadostat and other IDO inhibitors are listed in Table S1.

### 3.2. Co-Stimulatory Molecules

Co-stimulatory immune molecules promote T-cell activation and anti-tumor immunity, as well as shape the development of immune memory. As such, these molecules represent an attractive target to activate specific immune cell populations and promote anti-tumor immune activity. In particular, there exists significant opportunity to combine agonists of co-stimulatory molecules with PD-1 inhibitors in order to produce potent anti-tumor immune responses as well as potentially promote durable responses and immune memory.

4-1BB (CD137) is a member of the TNF receptor family which is expressed by several immune cells including activated T cells and NK cells. Binding of its ligand 4-1BBL promotes activation and proliferation of these cells leading to enhanced anti-tumor activity [34]. Additionally, 4-1BB activation is associated with the development of immune memory and represents an attractive target for the induction of durable immune responses. Two monoclonal antibodies that stimulate 4-1BB on T and NK cells have been evaluated in phase 1 trials with or without pembrolizumab. Urelumab is a non-ligand-blocking agonist which has been evaluated as monotherapy in a phase I clinical trial. This study demonstrated promising anti-tumor activity but a prohibitive level of immune-related hepatotoxicity potentially secondary to hyper-stimulation of 4-1BB secondary to antibody complex formation [35]. More recently, low-dose urelumab with PD-1 inhibitors has demonstrated reduced toxicity but disappointing activity amongst mNSCLC patients with only 1 of 34 patients achieving a response [36]. Utomilumab is a weaker agonist of 4-1BB found to exhibit a more favorable toxicity profile in early-phase studies although with limited activity as monotherapy [37]. A phase I study combining utomilumab with pembrolizumab demonstrated a response rate of 26% [38] and trials with other PD-1/L1 inhibitors are ongoing (Table S1).

OX40 (CD134) is another member of the TNF receptor family that promotes activation, survival and proliferation of effector T cells [7,39]. Several OX40 agonist antibodies have been investigated in phase 1 trials of solid organ tumors, as both monotherapy or in combination with anti-PD-1 or anti-CTLA-4 antibodies. Treatment has been reasonably tolerated but response rates have thus far been modest, between 7 and 13% in the combinations [40,41,42]. More work is needed to determine which patients will respond and which combinations will provide best response, and trials of other OX40 agents are ongoing (Table S1).

ICOS (CD278) is part of the CD28 co-receptor family and is expressed on activated T cells. It interacts with its ligand (ICOSL) to regulate effector and memory T-cell development, as well as the humoral immune response. It also has the capacity to trigger immunosuppressive activity through Tregs [19]. JTX-2011 (vopratelimab) was the first ICOS agonist antibody tested a phase 1 trial in advanced solid tumors as monotherapy or in combination with nivolumab. Treatment was tolerable but responses modest. A total of 3 of 34 combination patients had PR, and another 2 had stable disease [43]. A phase 2 study of vopratelimab in combination with ipilimumab in PD-1-treated mNSCLC and urothelial cancer was closed early due to failure to meet pre-specified interim criteria [44]. Another phase 2 trial of vopratelimab alone or in combination with a PD-1 inhibitor in treatment-naïve mNSCLC patients is ongoing (NCT04549025). A novel ICOS agonist antibody GSK3359609 is also being evaluated as part of a platform trial of novel agents in PD-1 refractory mNSCLC (NCT03739710), and separately in combination with tremelimumab (NCT03693612).

Despite the promise of combination immunotherapy with co-stimulatory molecule agonists, only modest clinical benefit has been reported to date. This can largely be attributed to both significant complexities involved in manipulating co-stimulatory molecules as well as significant associated toxicity risks. The rationale for therapeutically targeting co-stimulatory molecules to induce immune cell activation and overcome an immunosuppressive TIME remains strong, and ongoing trials may yet demonstrate promising results (Table S1).

## 4. Priming Strategies

Immune therapy combinations described thus far have focused on improving T-cell anti-tumor activity through the blockade of inhibitory checkpoints or the activation of costimulatory molecules. However, the absence of an effective T-cell-mediated immune response may occur due to a lack of T cells which have been primed to respond to specific tumor antigens, producing an absent or deeply dysfunctional immune infiltrate classically associated with a non-inflamed TIME. Several strategies have been proposed to enhance T-cell priming in an effort to generate an effective T-cell-mediated anti-tumor immune response, which may then be augmented by the addition of established checkpoint inhibitors.

### 4.1. Radiation

Radiation therapy is known to promote immune-modulatory effects within the TIME through a range of mechanisms [7]. Key among these is the induction of immunogenic cell death whereby tumor cell killing via ionizing radiation elicits immune activation through the release of damage-associated molecular pattern molecules (DAMPs) that are recognized by the innate immune system [45]. Rapid recruitment of dendritic cells and macrophages leads to increased antigen presentation of tumor-associated antigens and migration of T cells to the tumor [46]. Synergistic effects have been seen with the combination of radiation and checkpoint inhibition in preclinical and clinical studies [47,48].

The PACIFIC trial is perhaps the best-known radio-immunotherapy combination studied in NSCLC. Patients with unresectable stage III NSCLC who had not progressed following definitive chemoradiation were randomized to 12 months of PD-L1 inhibitor durvalumab or placebo. The 5 year outcomes were recently reported and the addition of durvalumab improved 60 month survival rates from 33% to 43% [49]. Subgroup analysis found that survival was higher in patients who commenced durvalumab within 14 days of radiation [50]. Recent small studies of pembrolizumab after SBRT in mNSCLC as well as following fractionated radiotherapy have also suggested enhanced clinical benefit from immunotherapy following radiotherapy [51,52].

Multiple trials of radiation in various combinations with PD-1 inhibitors, with or without chemotherapy, are proceeding across all stages of lung cancer (Table S1). There exists significant promise to utilize radiotherapy as a means of enhancing T-cell priming and immune activation in a fashion that may be particularly useful in immunologically cold or non-inflamed tumors particularly when utilized in combination with subsequent immune checkpoint inhibitors. A key outstanding question for the use of radiotherapy as a priming or combination immunotherapy strategy is the optimal timing and dosing for induction of an effective anti-tumor immune response as this may differ significantly from standard dosing utilized for ablative purposes.

### 4.2. STING Agonists

The stimulator of interferon genes (STING) pathway is an immune sensing pathway triggered by the presence of cytosolic DNA [53]. The STING pathway has been implicated in the danger response associated with radiation-induced immunogenic cell death as well as viral infections. DNA in the cytosol is detected by cyclic GMP-AMP synthase (cGAS) which activates STING, a protein located in the endoplasmic reticulum. Activated STING initiates a signaling cascade that results in transcription and production of inflammatory cytokines, promoting local DC maturation and activation, enhanced trafficking of immune cells, and CTL-mediating tumor killing (Figure 1) [54,55].

Based on preclinical observations of this pathway in action against implanted tumors, STING agonists have been explored as an alternative mechanism to create a more anti-tumorigenic TIME, that may then respond better to checkpoint inhibition. MIW815 (ADU-S100) was the first STING agonist to be tested clinically in a phase 1 trial of patients with advanced solid tumors or lymphoma. Patients received intratumoral injections of the agonist, in combination with intravenous anti-PD-1 spartalizumab [56]. It was well tolerated and demonstrated anti-tumor activity in PD-1-naïve triple-negative breast cancer and PD-1 refractory melanoma. Unfortunately, the study was discontinued by the sponsor due to limited clinical efficacy [57]. Another intratumoral STING agonist MK-1454 had minimal response when used as monotherapy in patients with advanced solid tumors, but 24% in combination with pembrolizumab [58]. Several other STING agonists or modulators of the STING pathway are being investigated (Table S1). Some of these can be administered more conveniently intravenously or intramuscularly, and an orally available STING agonist has demonstrated anti-tumor activity in preclinical models [59].

### 4.3. TLR Agonists

Another potential mechanism to activate the innate immune system is via Toll-like receptors, protein receptors on innate immune cells that recognize pathogen-associated and damage-associated molecular patterns (PAMPs and DAMPs). The role of TLRs in cancer, however, is complex and the specific TLR activated depends on the nature of the threat and the localization of the TLR. TLRs 1, 2, 5, 6 are located on the cell surface and predominantly respond to bacterial proteins, while TLR 3, 7, 8 and 9 are intracellular and are activated by foreign DNA taken up by the cell. Activated TLRs can promote production of pro-inflammatory cytokines, activation of APCs and induction of T-effector cells, but some can also promote angiogenesis and tumor invasion.

A range of TLR agonists have been developed but the complexity of the TLR role has been an obstacle. The most evidence to date has focused on TLR9 agonists, which are designed as analogues of the PAMP recognized by TLR9. These are largely administered via intratumoral or subcutaneous routes to enhance anti-tumor immune activity as well as limit systemic toxicity. A phase 1 study of TLR agonist tilsotolimod demonstrated moderate responses in heavily pretreated solid tumors and was reasonably tolerated [60]. Post-treatment biopsies revealed increased expression of multiple immune checkpoints, which suggests significant potential for combining their use with checkpoint inhibitors. A phase 1/2 study investigated the combination of tilsotolimod with ipilimumab in post-PD-1 treated melanoma. The disease control rate was 71%, with durable disease control [61]. However, the subsequent phase 3 trial did not meet its primary endpoint [62]. More trials are underway with other agonists of TLR9 and other TLRs in combination with checkpoint inhibitors (Table S1).

### 4.4. Oncolytic Viruses

Oncolytic virus (OV) therapy is another strategy aimed at promoting immune activation via targeted immunogenic cell death [63]. Oncolytic viruses are modified to preferentially infect tumor cells and their introduction into the TIME causes tumor cell lysis, release of DAMPs and PAMPs, activating innate immune sensors and recruitment of immune cells to drive an anti-tumor immune response [64,65].

The best studied OV is T-VEC (talimogene laherparepvec), a modified herpes simplex type 1 virus that contains a gene insertion encoding GM-CSF which further promotes recruitment and activation of APCs. It demonstrated significant responses in a phase 3 study when injected intratumorally in melanoma patients, leading to FDA approval [66,67]. The combination of OVs with checkpoint inhibitors is thought to be synergistic by creating and then sustaining an immunogenic, anti-tumor TIME. Early-phase studies combining T-VEC with ipilimumab or pembrolizumab in melanoma showed promising results [68,69]. A phase III study with T-VEC and pembrolizumab however was halted over futility at interim analysis. There have been limited studies to date of OVs in lung cancer. A phase II trial of TG4010, a modified vaccinia virus, in combination with nivolumab in metastatic NSCLC failed to meet its primary endpoint and further development has ceased [70]. CAVATAK, a modified coxsackievirus A21, in combination with pembrolizumab, demonstrated encouraging efficacy with response rates of 30% and was well-tolerated in a phase 1b study of patients with advanced NSCLC [71].

### 4.5. Cytokines

Inflammatory cytokines play a key role in T-cell priming as well as promoting and regulating cell-mediated immune responses. High-dose IL-2 monotherapy has been previously demonstrated to be able to induce durable clinical responses in melanoma as well as RCC but has been associated with significant toxicity which has limited its current use. The success of PD-1/PD-L1 inhibitors has generated renewed interest in the use of systemic inflammatory cytokines to augment the activity of immune checkpoint inhibitors as well as certain adoptive cell therapy strategies [72,73]. NKTR-214 (pegylated IL-2) combined with a PD-1 inhibitor is currently being evaluated in mNSCLC with early data suggesting potential activity of this combination as well as its potential to impact the composition of the TIME [74]. Additional inflammatory cytokine analogues such as IL-15 super-agonists are also currently in development [75,76].

## 5. VEGF, Targeted Therapy and Other Immunomodulators

### 5.1. VEGF

Multiple cytokines within the TIME play important roles in making it more immunosuppressive or immunostimulatory, and vascular endothelial growth factor (VEGF) and transforming growth factor beta (TGF-β) have both been studied as therapeutic targets. VEGF is predominantly involved in regulating angiogenesis, which is required for tumor growth; however, increasing evidence suggests that it also contributes to an immunosuppressive TIME. It acts via endothelial factors to physically prevent T-cell infiltration into the tumor, while suppressing dendritic cell maturation and promoting inhibitory Tregs and MDSCs [61,62,63]. TGF-β also mediates an immunosuppressive TIME through induction of Tregs, inhibition of Teff and promotion of angiogenesis and epithelial–mesenchymal transition, which are integral for tumor progression [77].

Anti-VEGF/VEGFR agents bevacizumab, ramucirumab and nintedanib have all demonstrated improved outcomes when combined with chemotherapy in treatment of lung cancer [78,79,80]. There is significant potential for greater response by using agents that inhibit VEGF-mediated immunosuppression to create a more immunogenic environment that allows for enhanced anti-tumor activity of checkpoint inhibitors [81]. The combination of chemoimmunotherapy and bevacizumab in IMpower150 demonstrated a survival benefit compared to chemotherapy and bevacizumab without atezolizumab, which supports the premise of a synergistic relationship between checkpoint blockade and bevacizumab [82].

Improved survival with the combination in IMpower150 was seen across all clinical subgroups, including patients with liver metastases and patients with EGFR and ALK driver mutations [50]. Liver metastases in NSCLC are a poor prognostic indicator and studies have previously shown limited benefit for this population with checkpoint-inhibitor monotherapy [83]. Patients with EGFR and ALK alterations who have failed first-line targeted therapy also do poorly with subsequent lines of therapy and trials have shown that outcomes with single-agent checkpoint inhibitors are not better than with standard chemotherapy [84]. The survival benefit with the IO-bevacizumab combination for these patient populations is encouraging and the precise mechanisms by which this is achieved are being explored. EGFR mutations and hepatocellular cancers are known to express high amounts of VEGF that make them more susceptible to VEGF inhibition, switching the TIME to a more immune-tolerant one susceptible to checkpoint blockade. More work is also being done investigating VEGFR and multi-targeted TKIs in combination with ICIs. Early-phase trials to date [85,86,87,88] have shown encouraging results but larger, randomized trials are awaited.

### 5.2. TGF-β

Trials with agents targeting TGF-β have been more challenging due to the complexities of TGF-β signaling pathways. One promising agent is bintrafusp alfa, a bifunctional fusion protein with a TGF-β “trap” fused to a PD-L1 monoclonal antibody. Concurrent inhibition of these two inhibitory pathways in a phase 1 study of patients with advanced NSCLC after progression on chemotherapy demonstrated favorable clinical activity, particularly in PD-L1 high tumors [89]. However, a phase 3 study randomizing PD-L1-positive mNSCLC patients to bintrafusp alpha or pembrolizumab failed to meet its primary endpoint [90]. Phase 2 studies in combination with chemotherapy and trials of other TGF-β targeting agents are ongoing (Table S1).

### 5.3. Targeted Therapy

The presence of specific driver mutations and co-mutations has been previously demonstrated to exert significant impact on the composition of the TIME in mNSCLC. This impact is perhaps most pronounced amongst KRAS mutant tumors where the presence of p53, LKB1 and KEAP1 co-mutations may produce drastically different TIME and likelihood of response to ICI [91]. This has led to significant interest in combining targeted therapies directed against targetable driver mutations with immunotherapy strategies. Early trials evaluating the combination of EGFR and ALK TKIs with PD1 inhibitors were largely curtailed by significant synergistic toxicity. The recent development of direct KRAS G12C inhibitors has renewed interest in the utility of combining targeted therapy with PD1 inhibitors particularly in classically PD-1 refractory KRAS-LKB1 and KRAS-KEAP1 co-mutated mNSCLC [92,93]. Multiple clinical trials are presently underway with combination arms evaluating G12C inhibitor and PD1 inhibitor strategies (Table S1).

## 6. Adoptive Cell Therapy

The strategies discussed so far have focused primarily on modifying the TIME through the stimulation of pre-existing native immune cells. An alternative strategy currently under investigation is adoptive cellular therapy (ACT), in which ex vivo expanded or engineered T cells are manufactured and directly infused into patients with the goal of increasing tumor-specific effector cells capable of driving a strong anti-tumor immune response [7].

Tumor-infiltrating lymphocyte (TIL) therapy involves the isolation of lymphocytes from an autologous tumor sample, ex vivo stimulation and expansion followed by infusion back into the patient [94]. Pre-infusion lymphodepletion removes competing and immunosuppressive cells, and post-infusion high-dose IL-2 promotes TIL activation and proliferation [95]. Early data suggest the potential for durable responses in melanoma patients [96]. A recently reported phase 1 study of TILs in combination with nivolumab in 20 patients with advanced anti-PD-1 resistant NSCLC demonstrated durable anti-tumor responses. Six out of 13 evaluable patients had confirmed radiographic response, and 2 of these had a complete response, which was ongoing at 1.5 years [97].

Engineered TCRs are an alternative approach to re-introduce T cells with receptors designed to target specific tumor neoantigen/MHC combinations. Engineered TCRs are inherently restricted to responding to antigens presented by specific HLA molecules which can both be difficult for patient selection as well as the potential for tumors to downregulate HLA expression as a mechanism of resistance [94]. Limitations secondary to HLA restriction can be potentially obviated through the use of chimeric antigen receptor T cells (CAR-T), which are synthetic hybrid receptors combining an extracellular antibody-derived receptor specific for tumor antigen with an intracellular activating domain [98]. Newer generations of CARs have been engineered to include costimulatory domains such as CD28 and 4-1BB to enhance and maintain T-cell response, and so-called “armored” CAR-T cells have been developed to secrete pro-inflammatory cytokines [94]. CD19 CAR-T cells have been hugely successful in treating relapsed /refractory leukemias and lymphomas [99,100]. The combination of ACT strategies with other immunotherapeutic strategies (e.g., ICI, cytokines) has significant potential to augment the activity and durability of adoptively transferred immune cell populations. Trials investigating the combination are ongoing in both hematological and solid tumors. Additional strategies utilizing bi-specific T-cell engagers (BiTEs) as well as novel tumor neo-antigen vaccination approaches are also in development (Table S1).

## 7. Conclusions

The success of PD-1 inhibitors has demonstrated the potentially transformational power of immunotherapy in mNSCLC. For the first time, we have seen that functional cures are now possible in mNSCLC albeit among a minority of patients. The challenge now facing us is the development of the next generation of rational immunotherapy combinations capable of ensuring durable long-term response and functional cure for a broader population. Careful consideration of the complex tumor–immune interactions that sculpt the TIME is key to understanding the factors and mechanisms that ultimately determine the success or failure of a given combination immunotherapy strategy. Importantly, a clear understanding of these mechanisms is key to developing rational biomarker-driven clinical trials of novel combination immunotherapeutic strategies. Multiple such promising combination strategies are currently in development with highly disparate targets and underlying mechanisms of action including co-inhibitory molecules, co-stimulatory molecules and a litany of immuno-modulatory and adoptive cell therapy strategies. In this context, there exists significant risk that a biomarker-agnostic “one-size-fits-all” approach to drug development with limited consideration for individual TIME characteristics will risk further high-profile failures of novel immunotherapy combinations. Furthermore, this approach risks inappropriately discarding promising combinations that may be active in defined subsets of patients but for whom any signal of activity is lost in a broad, non-biomarker defined study population. Careful consideration of the immunologic characteristics of mNSCLC patients will be essential to ensuring that the right combination immunotherapy is studied in a biomarker-driven trial focused on patients with a susceptible TIME. Significant potential exists for the myriad aforementioned combination immunotherapy strategies to transform the treatment of mNSCLC. However, the complexity of our approach to the development of the next-generation of immunotherapies must meet the complex challenges inherent in the immunobiology of NSCLC.

## Supplementary Materials

The following supporting information can be downloaded at: https://www.mdpi.com/article/10.3390/curroncol29010004/s1, Table S1. Ongoing or recently completed trials of immune checkpoint inhibitors (IO) in combination with novel targets for advanced non-small-cell lung cancer (NSCLC) [101,102,103,104,105,106,107,108,109,110,111,112,113,114,115,116,117,118,119,120,121,122,123,124,125,126,127,128,129,130,131,132,133,134,135,136,137,138,139,140,141,142,143,144,145,146].
