# Supplementary material for: New Strategies and Combinations to Improve Outcomes in Immunotherapy in Metastatic Non-Small-Cell Lung Cancer
Source: Curr Oncol. 2021 Dec 23;29(1):38–55. doi: 10.3390/curroncol29010004 (PMC8774728; doi:10.3390/curroncol29010004)
Supplement: Supplementary file 1 [file curroncol-29-00004-s001.zip › curroncol-1425617-supplementary.pdf]

Current Oncology

Supplementary Materials

New Strategies and Combinations to Improve Outcomes in Immunotherapy in Metastatic Non-Small-Cell Lung Cancer

Lucy Corke and Adrian Sacher

Table S1. Ongoing or recently completed trials of immune checkpoint inhibitors (IO) in combination with novel targets for advanced non-small-cell lung cancer (NSCLC).

Trial Identifier	Drug Combinations	Indication	Phase	Status
**Co-inhibitory checkpoints + IO**				
**CTLA-4**				
NCT02453282 (MYSTIC)	Durvalumab +/− tremelimumab vs. chemotherapy	First-line advanced NSCLC	III	Active, not recruiting [101]
NCT02542293 (NEPTUNE)	Durvalumab + tremelimumab vs. chemotherapy	First-line metastatic NSCLC	III	Active, not recruiting [102]
NCT03057106	Durvalumab + tremelimumab +/− chemotherapy	Metastatic NSCLC	II	Active, not recruiting [103]
NCT03164616 (POSEIDON)	Durvalumab (anti-PD-L1) + chemotherapy +/− tremelimumab	Treatment-naïve advanced NSCLC, EGFR/ALK wild type	III	Recruiting [104]
NCT03580694	REGN4659 (anti-CTLA4) + cemiplimab	Advanced NSCLC	I	Terminated [105]
NCT03515629 (EMPOWER-Lung 2)	Cemiplimab (anti-PD-1) + ipilimumab (anti-CTLA-4) +/− chemotherapy versus pembrolizumab	Treatment-naïve advanced NSCLC, PD-L1 > 50%, EGFR/ALK wild type	III	Completed
NCT03430063 (EMPOWER-Lung 4)	Cemiplimab +/− ipilimumab	Second-line advanced NSCLC, PD-L1 < 50%	II	Active, not recruiting [106]
NCT01454102 (CheckMate-012)	Ipilimumab + nivolumab (one of several nivolumab combination arms)	First-line advanced NSCLC	I	Active, not recruiting [107]
NCT02659059 (CheckMate-568)	Ipilimumab + nivolumab +/− chemotherapy	First-line advanced NSCLC	II	Active, not recruiting [108]
NCT03469960 (DICIPLE)	Ipilimumab + nivolumab continuous vs. induction and observation	Treatment-naïve advanced NSCLC, PD-L1 1–49%	III	Recruiting
NCT03529526	KN046 (PD-L1/CTLA-4 bi-specific antibody)	Advanced solid tumors	I	Active, not recruiting [109]
NCT03838848	KN046	Stage IV NSCLC	II	Recruiting
NCT03761017	MGD019 (PD-1/CTLA-4 bi-specific DART protein)	Unresectable/metastatic cancer	I	Recruiting [110]
NCT04140526	ONC-392 (anti-CTLA4) +/− pembrolizumab	Advanced solid tumors	I	Recruiting
NCT03302234 (KEYNOTE-598)	Pembrolizumab +/− ipilimumab	Untreated stage IV NSCLC	III	Active, not recruiting [17]

NCT02477826 (CheckMate 227)	Nivolumab vs. nivolumab + ipilimumab vs. nivolumab + chemotherapy	Chemotherapy-naïve stage IV NSCLC	III	Recruiting [15]
NCT03215706 (CheckMate 9LA)	Nivolumab + ipilimumab + chemotherapy vs. chemotherapy	First-line stage IV NSCLC	III	Active, not recruiting [14]
NCT04043195	Nivolumab + ipilimumab + oxaliplatin	Previously treated advanced NSCLC	I/II	Recruiting
NCT03179436	Quavonlimab + pembrolizumab	Advanced solid tumors	I/II	Recruiting [111]
NCT03516981 (KEYNOTE-495)	Quavonlimab + pembrolizumab (multiple arms of study)	Biomarker-directed combinations of pembrolizumab in advanced NSCLC	II	Recruiting
NCT04606472	SI-B003 (PD-1/CTLA-4 bi-specific antibody)	Advanced solid tumors	I	Recruiting
**TIGIT**				
NCT03260322	ASP8374 +/− pembrolizumab	Advanced solid tumors	I	Active, not recruiting
NCT02913313	BMS-986207 +/− nivolumab +/− ipilimumab	Advanced solid tumors	I /II	Recruiting
NCT05005273	BMS-986207 + nivolumab + ipilimumab	Treatment-naïve advanced NSCLC	II	Not yet recruiting
NCT04791839	Domvanalimab + zimerelimab (anti-PD1) + etrumadenat (adenosine receptor antagonist)	Previously treated advanced NSCLC, PD-L1 ≥1%	II	Recruiting
NCT04262856	Domvanalimab + zimerelimab +/− etrumadenat	Treatment-naïve advanced NSCLC, PD-L1 ≥1%	II	Recruiting
NCT04736173	Domvanalimab + zimerelimab vs. zimerelimab vs. chemotherapy	Treatment-naïve advanced PD-L1-positive NSCLC	III	Recruiting
NCT04746924	Ociperlimab + tislelizumab vs. pembrolizumab + placebo	Treatment-naïve advanced NSCLC, PD-L1 > 50%	III	Recruiting
NCT05014815	Ociperlimab + tislelizumab + chemotherapy	Treatment-naïve advanced NSCLC	II	Not yet recruiting
NCT03119428	OMP-313M32 +/− nivolumab	Advanced solid tumors	I	Terminated (sponsor decision)
NCT04254107	SEA-TGT + sasanlimab (subcutaneously administered anti-PD-1)	Advanced solid tumors	I	Recruiting
NCT02794571	Tiragolumab + atezolizumab	Advanced solid tumors	I	Recruiting[112]
NCT03563716	Tiragolumab + atezolizumab vs. placebo + atezolizumab	Treatment-naïve advanced NSCLC	II	Active, not recruiting [23]
NCT04294810 (SKYSCRAPER-01)	Tiragolumab + atezolizumab vs. placebo + atezolizumab	Treatment-naïve advanced NSCLC, PD-L1 high	III	Recruiting
NCT04619797 (SKYSCRAPER-06)	Tiragolumab + atezolizumab + chemotherapy vs. pembrolizumab + chemotherapy	Treatment-naïve advanced NSCLC	II	Recruiting
NCT04958811	Tiragolumab + atezolizumab + bevacizumab	Previously treated advanced non-squamous NSCLC	II	Not yet recruiting
NCT02964013	Vibostolimab +/− pembrolizumab	Advanced solid tumors	I	Recruiting
NCT04165070	Vibostolimab + pembrolizumab + chemotherapy (arms in umbrella study)	Treatment-naïve advanced NSCLC	II	Recruiting

NCT04738487	Vibostolimab/pembrolizumab coformulation vs. pembrolizumab alone	Treatment-naïve advanced NSCLC, PD-L1 ≥1%		
NCT04725188	Vibostolimab/pembrolizumab coformulation +/− docetaxel	Advanced NSCLC after treatment with chemotherapy/IO	II	Recruiting
**LAG-3**				
NCT03156114	BI 754111 +/− BI 754091 (anti-PD-1)	Advanced solid tumors	I	Active, not recruiting [113]
NCT03625323	Eftilagimod alpha (LAG-3 fusion protein) + pembrolizumab	Previously untreated advanced NSCLC, recurrent PD-X refractory NSCLC or HNSCC	II	Recruiting
NCT03252938	Eftilagimod alpha +/ avelumab or chemotherapy	Previously treated advanced solid tumors	I	Recruiting
NCT03516981 (KEYNOTE-495)	Favezelimab + pembrolizumab (multiple arms of study)	Biomarker-directed combinations of pembrolizumab in advanced NSCLC	II	Recruiting
NCT02720068	Favezelimab (MK-4280) +/− pembrolizumab +/− chemotherapy or lenvatinib	Advanced solid tumors	I	Recruiting
NCT02460224	LAG525 +/− spartalizumab	Advanced solid tumors	I/II	Completed [114]
NCT03005782	REGN3767 +/− cemiplimab	Advanced solid tumors	I	Recruiting [115]
NCT01968109	Relatlimab +/− nivolumab	Advanced solid tumors	I	Recruiting [116]
NCT03459222	Relatlimab + nivolumab or ipilimumab	Advanced solid tumors	I/II	Recruiting
NCT04623775	Relatlimab + nivolumab + chemotherapy vs. nivolumab + chemotherapy	Previously untreated advanced NSCLC	II	Recruiting
NCT03459222	Relatlimab + nivolumab + ipilimumab or IDO1 inhibitor	Advanced solid tumors	I/II	Recruiting
NCT04140500	RO7247669 (anti-PD1/anti-LAG-3 bispecific antibody)	Advanced solid tumors	I	Recruiting
NCT03219268	Tebotelimab (anti-PD1/anti-LAG-3 bi-specific DART molecule)	Unresectable or metastatic solid tumors	I	Active, not recruiting [117]
NCT03250832	TSR-033 + dostarlimab	Previously treated advanced solid tumors	I	Active, not recruiting
NCT02817633 (AMBER)	TSR-033 +/− anti-PD1, anti-TIM-3 (multiple intervention arms)	Advanced solid tumors	I	Recruiting
NCT03849469	XmAb22841 (anti-CTLA4/anti-LAG-3 bi-specific antibody) +/− pembrolizumab	Previously treated advanced solid tumors	I	Recruiting
**TIM-3**				
NCT03744468	BGB-A425 + tislelizumab	Advanced solid tumors	I/II	Recruiting
NCT03446040	BMS-986258 +/− nivolumab	Advanced solid tumors	I/II	Recruiting
NCT04370704	INCAGN02390 + anti-PD1, anti-LAG combinations	Advanced solid tumors	I/II	Recruiting
NCT03099109	LY3321367 + anti-PD1	Advanced relapsed/refractory solid tumors	I	Active, not recruiting [118]
NCT03311412	Sym023 + anti-PD1, anti-LAG combinations	Advanced solid tumor or lymphoma	I	Recruiting

NCT02817633 (AMBER)	TSR-022 +/− anti-PD1, anti-LAG, chemotherapy combinations (multiple intervention arms)	Advanced solid tumors	I	Recruiting
NCT04655976	TSR-022 (cobolimab) + dostarlimab	Advanced NSCLC, post-anti-PD1/PD-L1	II/III	Recruiting
NCT03708328	RO7121661 (anti-PD-1/antiTIM-3 bi-specific antibody)	Advanced solid tumors	I	Active, not recruiting
NCT02608268	Sabatolimab (MBG453) + spartalizumab	Advanced solid tumors	I/II	Active, not recruiting[119]
**IDO**				
NCT03417037	BMS-986205 + nivolumab +/− chemotherapy	First-line metastatic NSCLC	III	Withdrawn
NCT02658890	BMS-986205 + nivolumab +/− ipilimumab	Advanced cancers	I/II	Recruiting
NCT02862457 (KEYNOTE-434)	Epacadostat +/− pembrolizumab +/− chemotherapy	Advanced solid tumors	I	Completed [120]
NCT02298153 (ECHO-110)	Epacadostat + atezolizumab	Previously treated advanced NSCLC or UC	I	Terminated [121]
NCT02178722 (ECHO-202)	Epacadostat + pembrolizumab	Advanced solid tumors	I/II	Completed [122]
NCT02959437 (ECHO-206)	Epacadostat + pembrolizumab + azacitadine/other agents	Advanced solid tumors, including previously treated NSCLC cohort	I/II	Terminated
NCT03085914 (ECHO-207)	Epacadostat + pembrolizumab and chemotherapy	Advanced solid tumors	I/II	Completed
NCT03347123 (ECHO-208)	Epacadostat + nivolumab and ipilimumab or lirilumab	Advanced malignancies	I/II	Completed
NCT03322540 (ECHO-305)	Epacadostat + pembrolizumab vs. placebo + pembrolizumab	First-line metastatic NSCLC, PD-L1>50%	II	Initially phase III but due to results of ECHO-301 changed to proof of concept studies [123]
NCT03322566 (ECHO-306)	Epacadostat + pembrolizumab + chemotherapy vs. pembrolizumab + chemotherapy	First-line NSCLC	II	
NCT03348904 (ECHO-309)	Epacadostat + nivolumab + chemotherapy vs. chemotherapy	First-line metastatic NSCLC	III	Terminated
NCT03364049	MK-7162 + pembrolizumab	Advanced solid tumors	I	Completed
NCT02471846	Navoximod + atezolizumab	Advanced solid tumors	I	Completed [124]
**Co-stimulatory checkpoints + IO**				
**4-1BB**				
NCT03809624	INBRX-105 (4-1BB agonist /anti-PD-L1 bi-specific antibody) +/− pembrolizumab	Advanced solid tumors	I	Recruiting
NCT02534506	Urelumab + nivolumab	Advanced solid tumors	I	Completed
NCT01471210	Urelumab + nivolumab	Advanced solid tumors and relapsed/refractory B-cell lymphoma	I/II	Completed [36]
NCT03792724	Urelumab (intratumoral) + nivolumab	Advanced solid tumors	I/II	Not yet recruiting
NCT02179918	Utomilumab + pembrolizumab	Advanced solid tumors	I	Completed [38]
NCT02554812	Utomilumab + avelumab +/− OX40 agonist (multiple intervention arms)	Advanced solid tumors	II	Active, not recruiting

(JAVELIN medley)				
NCT03217747	Utomilumab + avelumab +/− OX40 agonist +/− radiation (multiple intervention arms)	Advanced solid tumors	I/II	Recruiting
**OX40**				
NCT04215978	BGB-A445 +/− tislelizumab	Advanced solid tumors	I	Recruiting
NCT02737475	BMS-986178 +/− nivolumab +/− ipilimumab	Advanced solid tumors	I/II	Completed [42]
NCT02528357	GSK3174998 +/− pembrolizumab	Advanced solid tumors	I	Completed [41]
NCT03758001	IBI101 + sintilimab	Advanced solid tumors	I	Active, not recruiting
NCT04198766	INBRX-106 (OX40 agonist) +/− pembrolizumab	Advanced solid tumors	I	Recruiting
NCT03241173	INCAGN01949 + nivolumab +/− ipilimumab	Advanced solid tumors	I/II	Completed
NCT02705482	MEDI0562 + durvalumab or tremelimumab	Advanced solid tumors	I	Completed [40]
NCT02221960	MEDI6383 +/− durvalumab	Advanced solid tumors	I	Completed
NCT02205333	MEDI6469 +/− durvalumab or tremelimumab	Advanced solid tumors	I/II	Terminated by sponsor
NCT02410512	MOXR0916 + atezolizumab	Advanced solid tumors	I	Completed [125]
NCT02554812 (JAVELIN medley)	PF-04518600 + avelumab +/− utomilumab (4-1BB agonist) (multiple intervention arms)	Advanced solid tumors	II	Active, not recruiting
NCT03217747	PF-04518600 + avelumab +/− utomilumab +/− radiation (multiple intervention arms)	Advanced solid tumors	I/II	Recruiting
**ICOS**				
NCT03251924	BMS-986226 +/− nivolumab or ipilimumab	Advanced solid tumors	I/II	Active, not recruiting
NCT02723955 (INDUCE-1)	GSK3359609 +/− pembrolizumab (multiple other intervention arms)	Advanced solid tumors	I	Active, not recruiting [126]
NCT03693612	GSK3359609 + tremelimumab	Advanced solid tumors	I/II	Active, not recruiting
NCT03739710	GSK3359609 + ipilimumab or dostarlimab (multiple intervention arms)	Previously treated advanced NSCLC	I/II	Recruiting
NCT02904226 (ICONIC)	Vopratelimab +/− nivolumab +/− ipilimumab	Advanced solid tumors	I/II	Completed [43]
NCT03989362	Vopratelimab + ipilimumab	Advanced NSCLC or urothelial cancer, previously treated with anti-PD-1/PD-L1	II	Active, not recruiting
NCT04549025	Vopratelimab + anti-PD1	Biomarker selected metastatic NSCLC	II	Recruiting
**Priming strategies + IO**				
**Radiation**				
NCT03223155	Concurrent or sequential SBRT + ipilimumab and nivolumab	Metastatic NSCLC	I	Recruiting
NCT03168464	Radiation + ipilimumab and nivolumab	Previously treated metastatic NSCLC	I/II	Recruiting

NCT03812549	Radiation + sintilimab	Treatment-naïve PD-L1 >1% metastatic NSCLC	I	Recruiting
NCT04878107	SBRT/LDRT + camrelizumab and apatinib or docetaxel	Metastatic NSCLC, post-chemo/IO	II	Not yet recruiting
NCT04238169	SBRT + toripalimab + bevacizumab	Previously treated non-squamous NSCLC	II	Recruiting
NCT03307759	SABR + pembrolizumab (before or after)	Metastatic NSCLC	I	Active, not recruiting
NCT02492568	SBRT +/− pembrolizumab	Previously treated advanced NSCLC	II	Completed [51]
NCT03212469	SBRT + durvalumab and tremelimumab	Metastatic squamous NSCLC, HNSCC, esophageal cancer	I/II	Recruiting
NCT03774732	Concurrent radiation + pembrolizumab + chemotherapy	Treatment-naïve advanced NSCLC	III	Recruiting
NCT02658097	Single fraction radiation + pembrolizumab	Previously treated advanced NSCLC	II	Active, not recruiting
NCT02444741	SBRT or wide-field radiation therapy +/− pembrolizumab	Metastatic NSCLC	II	Active, not recruiting [52]
NCT03867175	SBRT +/− pembrolizumab	Metastatic NSCLC	III	Recruiting
NCT03474497	Radiation + pembrolizumab + IL-2	Metastatic NSCLC, melanoma, RCC or HNSCC failed anti-PD-1/PD-L1 therapy	I/II	Recruiting
NCT04929041	Radiation + immunotherapy +/− chemotherapy	Metastatic NSCLC, PD-L1 negative	II/III	Not yet recruiting
NCT03391869	Local radiation +/− nivolumab and ipilimumab	Metastatic NSCLC	III	Recruiting
NCT02221739	Radiation + ipilimumab	Previously treated metastatic NSCLC	I/II	Completed [127]
NCT03044626	Radiation + nivolumab	Previously treated metastatic NSCLC	II	Completed [128]
NCT02696993	Radiation + nivolumab +/− ipilimumab	Advanced NSCLC with brain metastases	I/II	Recruiting
NCT02239900	SBRT + ipilimumab	Advanced solid tumors	I	Completed [129]
NCT03313804	Radiation + checkpoint inhibitor	NSCLC, HNSCC planned to commence checkpoint inhibitor monotherapy	II	Recruiting [130]
NCT03275597	SBRT + durvalumab and tremelimumab	Metastatic NSCLC	I	Active, not recruiting [131]
NCT03431948	SBRT + nivolumab or other immune-targeting agents	Metastatic solid tumors	I	Active, not recruiting
NCT02318771	Radiation + pembrolizumab	Advanced solid tumors	I	Active, not recruiting [132]
**STING agonists**				
NCT03172936	ADU-S100 + spartalizumab	Advanced solid tumors or lymphoma	I	Completed [56]
NCT02675439	ADU-S100 +/− ipilimumab	Advanced solid tumors or lymphoma	I	Active, not recruiting
NCT03956680	BMS-986301 +/− nivolumab and ipilimumab	Advanced solid tumors	I	Recruiting
NCT03843359	GSK3745417 +/− dostarlimab	Relapsed/refractory solid tumors	I	Recruiting

NCT03010176	MK-1454 +/− pembrolizumab	Advanced solid tumors or lymphomas		Active, not recruiting [58]
NCT04609579	SNX281 +/− pembrolizumab	Advanced solid tumors or lymphoma	I	Recruiting
NCT04167137	SYNB1891 +/− atezolizumab	Advanced solid tumors or lymphoma	I	Recruiting
NCT04420884	TAK-676 +/− pembrolizumab	Advanced solid tumors	I	Recruiting
NCT04879849	TAK-676 + pembrolizumab, following radiation	Advanced NSCLC, TNBC, HNCC	I	Recruiting
**TLR agonists**				
NCT03486301	BDB001 (TLR7/8) +/− pembrolizumab	Advanced solid tumors	I	Recruiting [133]
NCT04840394	BDB018 (TLR7/8) +/− pembrolizumab	Advanced solid tumors	I	Recruiting
NCT04101357	BNT411 (TLR7) +/− atezolizumab and chemotherapy	Refractory advanced solid tumors	I	Recruiting
NCT03438318	CMP-001 (TLR9) + atezolizumab +/− radiation	Advanced NSCLC, post-PD-1/PD-L1 treatment	I	Completed
NCT02554812 (JAVELIN medley)	CMP-001 + avelumab	Advanced solid tumors	II	Active, not recruiting
NCT03684785	Cavrotolimod (TLR9) + pembrolizumab or cemiplimab	Advanced solid tumors	I/II	Recruiting
NCT03416335	DSP-0509 (TLR7) +/− pembrolizumab	Advanced solid tumors	I/II	Recruiting
NCT03326752	DV281 (TLR9) + nivolumab	Advanced NSCLC	I	Completed [134]
NCT03447314	GSK1795091 (TLR4) + pembrolizumab (multiple other combination arms)	Advanced solid tumors	I	Active, not recruiting
NCT02668770	Lefitolimod (MGN1704, TLR9) + ipilimumab	Advanced solid tumors	I	Active, not recruiting
NCT03301896	LHC165 (TLR7) +/− spartalizumab	Advanced malignancies	I	Active, not recruiting [135]
NCT02556463	MEDI9197 (TLR7/8) + durvalumab	Solid tumors or cutaneous T-cell lymphoma	I	Terminated (sponsor strategy) [136]
NCT04588324	SHR2150 (TLR7) + pembrolizumab and chemotherapy	Advanced solid tumors	I/II	Recruiting
NCT04270864	Tilsotolimod (intratumoral) + ipilimumab (intratumoral) and nivolumab	Advanced solid tumors, includes anti-PD-1 refractory NSCLC cohort	I	Active, not recruiting
NCT04799054	TransCon (TLR7/8) +/− pembrolizumab	Refractory advanced solid tumors	I/II	Recruiting
NCT02643303	Poly-ICLC (TLR3) + durvalumab and tremelimumab	Advanced solid tumors	I/II	Completed
**Oncolytic viral therapy**				
NCT03004183	ADV/HSV-tk (oncolytic adenovirus) + valacyclovir + SBRT followed by pembrolizumab	Metastatic TNBC and metastatic NSCLC (1L or after platinum chemotherapy)	II	Active, not recruiting
NCT02831933	ADV/HSV-tk + valacyclovir + SBRT followed by nivolumab	Metastatic NSCLC (post-platinum/IO) or metastatic uveal melanoma	II	Terminated (lack of funding)
NCT04725331	BT-001 (Vaccinia virus) + pembrolizumab	Advanced solid tumors	I/II	Recruiting

NCT02043665	CVA21 (CAVATAK, coxsackievirus) +/− pembrolizumab	Advanced NSCLC and bladder cancer	I/Ib	Completed [71]
NCT04521621	V937 (CVA21, CAVATAK) + pembrolizumab	Advanced/metastatic solid tumors, no NSCLC cohort but any tumor with liver metastases	I/II	Recruiting
NCT02824965	CVA21 + pembrolizumab	Advanced NSCLC (refractory to 1 line of treatment)	I	Active, not recruiting
NCT02879760	MG1-MAGEA3 (Maraba virus) with Ad-MAGE3 (adenovirus) + pembrolizumab	Advanced NSCLC after 1 line of treatment	I/II	Completed
NCT03889275	MEDI5395 (Newcastle disease virus) + durvalumab	Advanced, refractory solid tumors	I	Recruiting
NCT05043714	NG-641 + nivolumab	Advanced epithelial tumors, including NSCLC, post-PD-1/PD-L1	I	Not yet recruiting
NCT03172819	OBP-301 (Telomelysin, adenovirus) + pembrolizumab	Advanced solid tumors	I	Active, not recruiting
NCT04386967	OH2 +/− pembrolizumab	Advanced solid tumors	I/II	Recruiting
NCT04348916	ONCR-177-101 + pembrolizumab	Advanced solid tumors	I	Recruiting
NCT02977156	Pexa-Vec (Vaccinia virus) + ipilimumab	Advanced solid tumors, refractory to treatment	I	Recruting
NCT03767348	RP1 (HSV-1) +/− nivolumab	Advanced solid tumors, includes post-anti-PD-1/PD-L1 failed NSCLC cohort	I/II	Recruiting
NCT04336241	RP2 + nivolumab	Advanced solid tumors	I	Recruiting
NCT02509507	T-VEC + pembrolizumab	Advanced solid tumors, includes NSCLC with liver metastases	I/II	Recruiting
NCT03597009	T-VEC + nivolumab	Advanced NSCLC with malignant pleural effusion	I/II	Terminated (slow accrual, funding withdrawal)
NCT02923466	VSV-IFNß-NIS +/− avelumab	Refractory advanced solid tumors	I	Active, not recruiting [137]
NCT03647163	VSV-IFNß-NIS + pembrolizumab	Refractory HNSCC and NSCLC	I/II	Recruiting
NCT04291105	VV1 (VSV-IFNß-NIS) + cemiplimab	Advanced solid tumor, includes NSCLC cohort	II	Recruiting
**Cytokines**				
NCT02523469	ALT-803 (IL-15 superagonist) + nivolumab	Pretreated advanced NSCLC	I/II	Active, not recruiting [76]
NCT03228667	ALT-803 + checkpoint inhibitor	Checkpoint relapsed advanced solid tumors	II	Active, not recruiting [75]
NCT03474497	IL-2 + pembrolizumab + radiation	Advanced solid tumors	I/II	Recruiting
NCT0279909 (ARTISTRY-1)	Nemvaleukin alfa (engineered IL-2) +/− pembrolizumab	Advanced solid tumors	I/II	Active, not recruiting [73]
NCT02983045 (PIVOT-2)	NKTR-214 (pegylated IL-2) + nivolumab +/− ipilimumab	Advanced solid tumors	I/II	Active, not recruiting [74]
NCT03138889	NKTR-214 + pembrolizumab +/− chemotherapy	Advanced solid tumors	I/II	Recruiting

**VEGF, targeted and immunomodulatory agents + IO**				
**VEGF/VEGFR-directed therapy**				
NCT04164745	Anlotinib + pembrolizumab	Treatment-naïve advanced NSCLC, PD-L1≥1%	II	Recruiting
NCT04165330	Anlotinib + nivolumab	Advanced solid tumors	I/II	Recruiting
NCT04211896	Anlotinib + nivolumab	Second-line advanced NSCLC	II	Not yet recruiting
NCT04507906	Anlotinib + nivolumab	Advanced NSCLC, previously treated with checkpoint inhibitor	II	Recruiting
NCT04124731	Anlotinib + sintilimab (anti-PD1)	Treatment-naïve advanced NSCLC	II	Not yet recruiting
NCT04846452	Anlotinib + sintilimab + chemotherapy	Treatment-naïve advanced NSCLC	II	Recruiting
NCT04790409	Anlotinib + sintilimab	Advanced NSCLC with uncommon mutations	II	Recruiting
NCT03765775	Anlotinib + sintilimab	Advanced EGFR mutation-positive NSCLC after first-generation TKI, T790M negative		Recruiting
NCT04316351	Anlotinib + toripalimab (anti-PD1) + pemetrexed	Advanced EGFR T790M mutation-positive NSCLC following osimertinib	II	Recruiting
NCT04239443	Apatinib + camrelizumab	Advanced NSCLC, uterine cancer, soft tissue sarcoma	II	Recruiting
NCT04670913	Apatinib + camrelizumab	Advanced NSCLC previously treated with first-line immunotherapy	II	Recruiting
NCT04459078	Apatinib + camrelizumab and albumin paclitaxel	Metastatic lung adenocarcinoma	II	Not yet recruiting
NCT04203485	Apatinib + camrelizumab vs. camrelizumab alone	Treatment-naïve advanced PD-L1-positive NSCLC	III	Not yet recruiting
NCT03777124	Apatinib + camrelizumab vs. chemotherapy	Metastatic non-squamous KRAS mutant NSCLC	II	Not yet recruiting
NCT03472560 (JAVELIN Medley VEGF)	Axitinib + avelumab	Advanced NSCLC or urothelial cancer	II	Active, not recruiting [138]
NCT03386929	Axitinib + avelumab + palbociclib	Advanced NSCLC with no driver mutations	I/II	Active, not recruiting [139]
NCT04585815	Axitinib + sasanlimab (anti-PD1) + SEA-TGT (anti-TIGIT)	Advanced NSCLC	I/II	Recruiting
NCT04563338 (INTEGRATE)	Bevacizumab + atezolizumab	Advanced NSCLC with liver metastases and PD-L1 > 50%; HCC	II	Recruiting
NCT04099836	Bevacizumab + atezolizumab	Advanced EGFR mutation-positive NSCLC after failure of osimertinib	II	Recruiting
NCT03836066	Bevacizumab + atezolizumab	Treatment-naïve NSCLC with high TMB	II	Active, not recruiting
NCT03896074	Bevacizumab + atezolizumab vs. atezolizumab alone	Treatment-naïve NSCLC, PD-L1 ≥ 1%	II	Recruiting

NCT03991403	Bevacizumab + atezolizumab + chemotherapy vs. chemotherapy alone	Previously treated EGFR or ALK mutation-positive advanced NSCLC	III	Recruiting
NCT04042558	Bevacizumab + atezolizumab + chemotherapy	Previously treated EGFR, ALK or ROS1 mutation-positive advanced NSCLC	II	Recruiting
NCT04147351	Bevacizumab + atezolizumab + chemotherapy	Previously treated EGFR mutation-positive advanced NSCLC	II	Recruiting
NCT04245085	Bevacizumab + atezolizumab + platinum doublet chemotherapy or pemetrexed alone	Previously treated EGFR mutation-positive advanced NSCLC	II	Recruiting
NCT04958811	Bevacizumab + atezolizumab + tiragolumab	Previously treated non-squamous NSCLC	II	Not yet recruiting
NCT03713944	Bevacizumab + atezolizumab + chemotherapy	Treatment-naïve advanced non-squamous NSCLC	II	Active, not recruiting [140]
NCT02366143 (IMpower150)	Bevacizumab + atezolizumab + chemotherapy vs. bevacizumab + chemotherapy vs. atezolizumab + chemotherapy	Treatment-naïve advanced non-squamous NSCLC	III	Completed [82]
NCT04194203 (IMpower151)	Bevacizumab + chemotherapy +/− atezolizumab	Treatment-naïve advanced non-squamous NSCLC	III	Recruiting
NCT02681549	Bevacizumab + pembrolizumab	Previously treated melanoma or NSCLC with new brain metastasis	II	Recruiting
NCT04517526	Bevacizumab + durvalumab + chemotherapy + SBRT	Previously treated EGFR mutation-positive advanced NSCLC after failure of osimertinib	II	Not yet recruiting
NCT03802240	IBI305 (bevacizumab biosimilar) + sintilimab + chemotherapy	Advanced non-squamous NSCLC with driver mutation, failed first-line TKI	III	Recruiting
NCT03516981 (KEYNOTE-495)	Lenvatinib + pembrolizumab (multiple arms of study)	Biomarker-directed combinations of pembrolizumab in advanced NSCLC	II	Recruiting
NCT03829319 (LEAP-006)	Lenvatinib + pembrolizumab + chemotherapy	Treatment-naïve advanced non-squamous NSCLC	III	Active, not recruiting [141]
NCT03829332 (LEAP-007)	Lenvatinib + pembrolizumab	Treatment-naïve advanced NSCLC, TPS ≥ 1%	III	Active, not recruiting
NCT03976375 (LEAP-008)	Lenvatinib + pembrolizumab vs. docetaxel	Advanced NSCLC after progression on platinum/IO	III	Recruiting
NCT04989322	Lenvatinib + pembrolizumab + chemotherapy	Advanced NSCLC with driver mutation, failed first-line TKI	II	Not yet recruiting
NCT05024214	Lenvatinib + envafolimab (anti-PD-1)	Advanced solid tumors	I/II	Not yet recruiting
NCT04777084	Lenvatinib + IPI318 (anti-PD-1/PD-L1 antibody)	Advanced NSCLC after failure of anti-PD-1/L1	II	Recruiting
NCT05001724	Lenvatinib + KN046 (PD-L1/CTLA-4 bi-specific antibody)	Advanced NSCLC after failure of anti-PD-1/L1	II/III	Not yet recruiting

NCT03377023	Nintedanib + nivolumab + ipilimumab	Treatment-naïve or IO pretreated advanced NSCLC	I/II	Recruiting
NCT03689855	Ramucirumab + atezolizumab	Advanced NSCLC after progression on checkpoint blockade	II	Active, not recruiting
NCT02572687	Ramucirumab + durvalumab	Advanced thoracic or gastrointestinal malignancies	I	Completed [142]
NCT02443324	Ramucirumab + pembrolizumab	Advanced NSCLC; urothelial, biliary tract or gastric cancers	I/II	Active, not recruiting [143,144]
NCT04120454	Ramucirumab + pembrolizumab	EGFR mutant recurrent or metastatic NSCLC	II	Recruiting
NCT04340882 (TaxRemPem)	Ramucirumab + pembrolizumab + docetaxel	Advanced NSCLC, post-platinum/IO	II	Recruiting
NCT03527108	Ramucirumab + nivolumab	Advanced NSCLC	II	Recruiting
NCT04696848	CDK516 (novel vascular disrupting agent) + durvalumab	Refractory solid tumors	I/II	Recruiting
**TGF-β**				
NCT02517398	Bintrafusp alfa (fusion protein with TGF-β trap and anti-PD-L1)	Previously treated NSCLC	I	Active, not recruiting [89]
NCT03631706	Bintrafusp alfa vs. pembrolizumab	1L advanced NSCLC, PD-L1 high	III	Discontinued by sponsor [90]
NCT03840915	Bintrafusp alfa + various chemotherapy regimens	1L or 2L advanced NSCLC	Ib/II	Active, not recruiting
NCT04971187	Bintrafusp alfa + platinum-pemetrexed	Advanced NSCLC with EGFR mutation after 1L TKI	II	Recruiting
NCT04396535	Bintrafusp alfa, docetaxel	Advanced NSCLC after platinum/IO	II	Recruiting
NCT02423343	Galunisertib + nivolumab	Advanced solid tumors, recurrent or refractory NSCLC	Ib/II	Completed
NCT03192345	SAR439459 +/− cemiplimab	Advanced solid tumors, includes post-PD-1/PD-L1 NSCLC cohort	I/Ib	Recruiting
NCT04291079	SRK-181 +/− anti-PD-(L)-1	Advanced solid tumors, includes post-PD-1/PD-L1 NSCLC cohort	I	Recruiting
NCT03732274	Vactosertib + durvalumab	Advanced NSCLC after platinum chemotherapy	Ib/IIa	Active, not recruiting
NCT04515979	Vactosertinb + pembrolizumab	1L NSCLC, PD-L1 ≥ 1%	II	Recruiting
**KRASG12C**				
NCT04449874	GDC-6036 +/− atezolizumab or other agents	Advanced solid tumors with KRAS G12C mutation	I	Recruiting
NCT04699188	JDQ443 +/− spartalizumab	Advanced solid tumors with KRASG12C mutation	I/II	Recruiting
NCT03785249 (KRYSTAL-1)	MRTX849 +/− pembrolizumab or other agents	Advanced solid tumors with KRASG12C mutation	I/II	Recruiting
NCT04613596 (KRYSTAL-7)	MRTX849 + pembrolizumab	Advanced NSCLC with KRASG12C mutation	II	Recruiting
**Adoptive cell therapy**				

NCT03907852	Gavo-cel + anti-PD1	Advanced mesothelin-expressing cancer, includes NSCLC	I/II	Recruiting
NCT02414269	iCasp9M28z T-cell infusions + pembrolizumab	Malignant pleural disease associated with NSCLC, mesothelioma or breast cancer	I/II	Recruiting
NCT02876510	IMA101 + atezolizumab	Relapsed/refractory advanced solid tumors	I	Active, not recruiting[145]
NCT03709706	Lete-cel +/− pembrolizumab	Advanced or recurrent NSCLC, HLA-A2+ with NY-ESO-1- or LAGE-1a-positive	I/II	Recruiting
NCT03645928	Lifileucel (LN-145) +/− pembrolizumab or ipilimumab and nivolumab	Advanced solid tumors, including previously treated NSCLC cohorts	II	Recruiting
NCT04639245	MAGE-A1-specific T-cell-receptor-transduced autologous T cells + atezolizumab	Previously treated metastatic NSCLC	I/II	Recruiting
NCT03215810	TIL + nivolumab	Advanced NSCLC, naïve to PD-1/PD-L1	I	Active, not recruiting [97]
NCT03296137	TIL + ipilimumab and nivolumab	Advanced solid tumors	I/II	Active, not recruiting [146]
